# Facile Chemical Synthesis of Doped ZnO Nanocrystals Exploiting Oleic Acid

**DOI:** 10.3390/nano10061150

**Published:** 2020-06-11

**Authors:** Sugata Barui, Roberto Gerbaldo, Nadia Garino, Rosaria Brescia, Francesco Laviano, Valentina Cauda

**Affiliations:** 1 Department of Applied Science and Technology, Politecnico di Torino, Corso Duca degli Abruzzi 24, 10129 Turin, Italy; sugata.barui@polito.it (S.B.); roberto.gerbaldo@polito.it (R.G.); nadia.garino@polito.it (N.G.); francesco.laviano@polito.it (F.L.); 2 Electron Microscopy Facility, Center for Convergent Technologies, Istituto Italiano di Tecnologia (IIT), via Morego 30, 16163 Genoa, Italy; rosaria.brescia@iit.it

**Keywords:** doped ZnO nanocrystals, Gd/Mn doping, oleate-stabilized, coprecipitation, optical and magnetic properties

## Abstract

Zinc oxide nanocrystals (ZnO-NCs) doped with transition metal elements or rare earth elements can be probed for magnetic resonance imaging to be used as a molecular imaging technique for accurate diagnosis of various diseases. Herein, we use Mn as a candidate of transition metal elements and Gd as a presenter of rare earth elements. We report an easy and fast coprecipitation method exploiting oleic acid to synthesize spherical-shaped, small-sized doped ZnO-NCs. We show the improved colloidal stability of oleate-stabilized doped ZnO-NCs compared to the doped ZnO-NCs synthesized by conventional sol–gel synthesis method, i.e., without a stabilizing agent, especially for the Mn dopant. We also analyze their structural, morphological, optical, and magnetic properties. We are able to characterize the persistence of the crystalline properties (wurtzite structure) of ZnO in the doped structure and exclude the formation of undesired oxides by doping elements. Importantly, we determine the room-temperature ferromagnetism of the doped ZnO-NCs. This oleate-stabilized coprecipitation method can be subjected as a standard procedure to synthesize doped and also co-doped ZnO-NCs with any transition metal elements or rare earth elements. In the future, oleate-stabilized Gd/Mn-doped ZnO-NCs can be exploited as magnetic resonance imaging (MRI) contrast agents and possibly increase the signal intensity on T1-weighted images or reduce the signal intensity on T2-weighted images.

## 1. Introduction

Among the semiconducting metal oxide nanoparticles, zinc oxide (ZnO) has a wide range of potential applications in many areas, such as optoelectronic devices, solar cells, chemical sensor, and photocatalysis. These manifold applications can be reconducted to the direct wide-band gap of about 3.3 eV, the light absorption properties in the ultraviolet (UV) region, and the large exciton binding energy of about 60 meV of ZnO nanomaterials [1,2,3]. Moreover, the use of ZnO nanocrystals (NCs) in biomedical applications is gaining increasing importance thanks to their anticancer, antibacterial, antioxidant, and anti-inflammatory activities; drug delivery capabilities; and remarkable optical properties to be used for nanoimaging or even diagnosis [2,4,5,6,7]. Even though ZnO-NCs have a broad range of applications, certain limitations are associated with their light absorption and photoexcitation or photocatalytic activities [8]. Due to the broad band gap at room temperature, as stated above, pure ZnO can only absorb light within the UV region. Another factor contributing to the limitation of photocatalytic or photoexcitation activity is the fast recombination rate of photogenerated electron–hole pairs [3]. Hence, to improve the above-mentioned activities, the absorption spectrum of ZnO needs to be addressed, from the UV range to the visible light region. In this way, more electron–hole pairs can be photogenerated and their lifetimes can be increased. Additionally, imposing magnetic behavior on the ZnO-NCs would result useful in the direction of bioimaging applications [9]. Doping of ZnO with magnetic transition metals such as Mn, Ni, Fe, Co, Sb, and Cr was introduced with the aim of tuning the optical, magnetic, electrical, or piezoelectric properties of ZnO [10,11,12,13,14,15,16]. Actually, the term “doped” stands for incorporating impurities, i.e., ions, in the host lattice and eventually modifying the properties of the host, as mentioned above. It has been reported that the ZnO doped with transition metals develops ferromagnetic properties at room temperature and might act as a high Curie temperature diluted magnetic semiconductor (DMS) [9,17]. As the transition metal dopant ions are ferromagnetically coupled with metal oxide, these ions contribute to spin polarization of the charge carriers of the semiconductor. Among the transition elements, Mn possesses maximal magnetic behaviors, having an effective electron mass of ~0.3 m_e_ (m_e_ = free electron mass). The doping of Mn into the ZnO semiconductor host lattice may allow increases of the injected spins and carriers to make it appropriate and useful as a DMS [18,19]. In fact, the easy overlapping of the d electron of Mn at the t_2g_ level with the ZnO valence band distinguishes Mn as the preferred dopant for ZnO compared to other transition elements. This is why we chose Mn as a candidate for transition metal elements to synthesize doped ZnO-NCs.

On the other hand, it has been demonstrated that ZnO doped with rare-earth ions can also create ferromagnetism at room temperature [20]. The magnetic property of the transition metal or rare earth elements is due to the presence of unpaired electrons in the outermost 3d or 4f orbitals, respectively [21]. Actually, these unpaired electrons possess magnetic moments and contribute to the room-temperature ferromagnetism, although the nature of the magnetic moments of transition metals and rare earth elements are quite different. For the transition metal ions, the outermost 3d electrons are delocalized and lead to strong direct exchange interactions and high Curie temperatures. However, these metals have a small total magnetic moment per atom, as their orbital momentum is frequently zero. For the rare earth metals, the 4f electrons in the periphery are localized and exchange interactions occur among 5d or 6s conduction electrons. These electrons are indirect and have high orbital momentum; as a result, they possess high total magnetic moments per atom [22,23]. Additionally, several studies have depicted the room-temperature ferromagnetism of Gd-doped ZnO nanoparticles [24,25]. To this end, we selected Gd as a presenter of the rare-earth elements to dope ZnO-NCs and compare their optical and magnetic properties with respect to Mn-doped ZnO-NCs.

It has been reported that the magnetic spins of the dopant ions should be localized on uniform sites in the host material to ensure they are ferromagnetically aligned through an indirect kind of magnetic coupling or through a carrier medium [26]. Basically, the local structure and ferromagnetic properties of the doped ZnO are strongly correlated to the synthetic parameters [27]. Currently, there are various methods that can be used to synthesize Gd/Mn-doped ZnO nanoparticles, such as wet chemical, sol–gel, coprecipitation, and hydrothermal approaches, among others. In order to improve the optical and magnetic properties, the search for an efficient synthesis method to prepare doped ZnO-NCs with a spherical shape and small particle size has become a major challenge for the researchers. Herein, we report on two different synthesis methods: one based on the conventional sol–gel synthesis and the other on the oleic-acid-based coprecipitation method. The samples are then thoroughly examined by different characterization techniques, including dynamic light scattering and Z-potential measurements, transmission and field-emission scanning electron microscopies coupled to energy dispersive spectroscopy, X-ray diffraction, X-ray photoelectron spectroscopy, UV–Vis absorption spectroscopy, and DC magnetization measurements. Thus, we compare the structural, morphological, optical, and magnetic properties of Gd/Mn-doped ZnO-NCs synthesized by the two different methods. We also show here that the sol–gel synthesis is very slow and does not work well for the Mn dopant, whereas the reaction rate for the coprecipitation method exploiting oleic acid is very fast. This allows the process time to be reduced from several hours for the sol–gel method to several minutes for the coprecipitation method using oleic acid. This synthesis method works efficiently for both dopants, allowing spherical small-sized doped ZnO-NCs to be obtained. We exploit oleic acid in the chemical synthesis of doped ZnO-NCs, as it has already been reported that it can strongly coordinate with ZnO by forming zinc oleate and provide a ligand passivation shell on the surface of ZnO-NCs. We demonstrate here that oleic acid not only stabilizes the NCs against aggregation, but also controls the size and shape of the NCs [28,29,30,31]. Herein, we report for the first time on the synthesis and characterization of oleate-stabilized Mn-doped ZnO-NCs.

## 2. Materials and Methods

### 2.1. Materials

Zinc acetate dihydrate (Zn(CH_3_COO)_2_·2H_2_O, ACS Reagent, ≥99.0%), gadolinium acetate hydrate (Gd(CH_3_COO)_3_·H_2_O, 99.9% trace metals basis), manganese acetate tetrahydrate (Mn(CH_3_COO)_2_·4H_2_O, ACS reagent, ≥98%), sodium hydroxide (NaOH pellets, ACS Reagent, ≥97.0%), tetramethylammonium hydroxide (TMAH) pentahydrate (98.5%), and oleic acid (≥99%) were purchased from Sigma-Aldrich (Darmstadt, Germany). Methanol (Ph Eur Grade VWR Chemicals, Radnor, PA, USA) and ethanol (Sigma-Aldrich, 99%) were used as solvents. We used double-distilled water direct from a Q3 system (Millipore) along with all the chemicals as purchased, without doing further purification.

### 2.2. Sol–Gel Synthesis of Undoped and Gadolinum and Manganese Doped ZnO-NCs (ZnO, ZnO–GdX, ZnO–MnX)

Sol–gel synthesis of undoped ZnO-NCs was carried out by following the conventional method as described in Dumontel et al. [32]. Briefly, Zn(CH_3_COO)_2_·2H_2_O (0.818 g, 3.73 mmol) was taken in a 100 mL round-bottom flask and dissolved in methanol (42 mL). The solution was heated to 60 °C under constant stirring, and after reaching the temperature at 60 °C, double-distilled water (318 µL) was added. Then, the solution of NaOH (0.289 g, 7.22 mmol) in methanol (23 mL) was added dropwise to the zinc acetate solution for about 15 min. Finally, the reaction mixture was stirred at 60 °C for 2.15 h and then washed with ethanol twice by performing a centrifugation and redispersion processes.

To synthesize Gd/Mn-doped ZnO-NCs, a similar method was used as described above. Along with Zn(CH_3_COO)_2_·2H_2_O (0.818 g, 3.73 mmol), either Gd(CH_3_COO)_3_·H_2_O (to synthesize Gd-doped ZnO-NCs) or Mn(CH_3_COO)_2_·4H_2_O (to synthesize Mn-doped ZnO-NCs) was dissolved in 42 mL of methanol and heated to 60 °C under constant stirring. Then, 318 µL of double-distilled water and 23 mL of methanolic NaOH solution (0.289 g, 7.22 mmol) were added as mentioned above. Finally, the reaction mixture was stirred at 60 °C for 6 h for Gd doping and 18 h for Mn doping, and then washed with ethanol for twice by performing centrifugation and redispersion processes. The mole ratios of Zn(CH_3_COO)_2_·2H_2_O and Gd(CH_3_COO)_3_·H_2_O/Mn(CH_3_COO)_2_·4H_2_O were 1:0.06, 1:0.12, and 1:0.24 (here, X represents 0.06, 0.12, or 0.24).

### 2.3. Coprecipitation Method to Synthesize Undoped and Doped Oleate-stabilized ZnO-NCs (Ol-ZnO, Ol-ZnO–GdX, Ol-ZnO–MnX)

To synthesize undoped or doped oleate-stabilized ZnO-NCs, a coprecipitation method was used, which was similar to that described by Yin et al. [33]. Briefly, Zn(CH_3_COO)_2_.2H_2_O (0.5268 g, 2.4 mmol), either alone or with Gd(CH_3_COO)_3_·H_2_O (for Gd doping)/Mn(CH_3_COO)_2_·4H_2_O (for Mn doping), was dissolved in 40 mL of absolute ethanol in a 100-mL round-bottom flask. Then, 140 µL of oleic acid was added and the reaction mixture was heated to 70 °C under constant stirring. After reaching the temperature, a TMAH solution (0.522 g of TMAH dissolved first in 1.052 mL of double-distilled water and then added to 10 mL of absolute ethanol) was rapidly transferred into the reaction mixture under vigorous stirring. After stirring for 10 min, 50 mL of ice-cold absolute ethanol was added and the reaction vessel was immediately put in an ice bath for further cooling. The resulted undoped and doped oleate-stabilized ZnO-NCs were collected by centrifugation and then washed with ethanol twice by performing centrifugation and redispersion processes. The mole ratios of Zn(CH_3_COO)_2_·2H_2_O and Gd(CH_3_COO)_3_·H_2_O/Mn(CH_3_COO)_2_·4H_2_O were 1:0.06, 1:0.12, and 1:0.24 (here, X represents 0.06, 0.12, or 0.24).

### 2.4. Characterization

The hydrodynamic sizes and polydispersity indexes (PDI) of the nanocrystals in ethanol and water were evaluated using the dynamic light scattering (DLS) technique with a Zetasizer Nano ZS90 (Malvern Instruments, Worcestershire, UK). Zeta potential values of the NCs were also determined in neutral water at pH 7 using the same instrument. All the DLS and Zeta potential measurements were carried out at room temperature at a concentration of 100 µg/mL and each sample was sonicated for 10 min before the acquisition.

To evaluate the overall quality, tentative size, and shape of the synthesized NCs, field emission scanning electron microscopy (FESEM, Merlin, ZEISS, Jena, Germany) was used. Elemental analysis of the materials was evaluated by using energy dispersive X-ray spectroscopy (EDS). The samples were prepared by dropping of the diluted NC solution in ethanol on top of a silicon wafer.

Additionally, to confirm the detailed size, composition, morphological, and structural features of the different materials, high-resolution transmission electron microscopy (HRTEM), high-angle annular dark field-scanning TEM (HAADF-STEM) imaging, and scanning TEM coupled to EDS (STEM-EDS) were used. Each sample was drop-cast onto a double amorphous carbon film (ultrathin carbon on holey carbon)-coated Cu grid. HAADF-STEM, HRTEM, and STEM-EDS analyses were carried out on a JEM-2200FS TEM (Schottky emitter, JEOL Ltd., Tokyo Japan), operated at 200 kV, equipped with a C_S_ corrector (CEOS GmbH, Heidelberg, Germany) for the objective lens, an in-column image filter (Ω-type), and a Bruker XFlash 5060 EDS silicon drift detector. To evaluate the size of nanocrystals, statistical measurements were done on the longest diameter (Feret’s diameter) of NCs in HRTEM images, over around 10 NCs per sample. For EDS quantification, the Cliff–Lorimer method was used by considering the K series of O, Mn, and Zn, and the L series for Gd.

Furthermore, to verify the presence of the oleate group and confirm the success of the coprecipitation method in synthesizing undoped and doped oleate-stabilized ZnO-NCs, the Fourier transform infrared spectra (FT-IR) were recorded in transmission mode with a Bruker Equinox 55 spectrometer (Bruker, Billerica, MA, USA) in the region of 4000–400 cm^−1^.

XRD (X-ray diffraction) analysis was performed at room temperature to investigate the crystalline structure of the prepared materials by using a Panalytical X’Pert diffractometer in *θ*–2*θ* Bragg–Brentano configuration equipped with a source of Cu-Kα radiation (*λ* = 1.54 Å, 40 kV and 30 mA). The samples were prepared by depositing the colloidal solution drop-by-drop on a silicon wafer. All the XRD analyses were carried out in the 2*θ* range of 20°–65°, with a step size of 0.02° (2*θ*) and an acquisition time of 300 s per step. The full width at half maximum (FWHM) values were calculated by using multiple peak fitting functions in Origin software, selecting Gauss as the peak function. The average crystallite size of each sample was calculated from the FWHM of the strongest reflection (101) peak by means of the Debye–Scherrer equation, *D* = kλ/*β*cos*θ*, where *θ* is the diffraction angle, *β* is the FWHM of the diffraction peak, k is the geometric factor (~0.9), λ is the X-ray wavelength used, and D is the mean crystallite size.

For further evaluation of the chemical composition and surface of the synthesized materials, X-ray photoelectron spectroscopic (XPS) analysis was performed using a PHI 5000 Versaprobe Scanning X-ray photoelectron spectrometer (monochromatic Al K-alpha X-ray source with 1486.6 eV energy) from Physical Electronics, Inc. (Chanhassen, MN, USA). To collect the photoelectron signal for both the high-resolution (HR) and survey spectra, a spot size of 100 µm was used.

The optical properties of the samples were evaluated by UV–Vis spectrophotometry. UV–visible absorbance spectra of the NCs (in ethanolic suspension at a concentration of 0.5 mg/mL) were recorded using a Multiskan GO microplate UV–Vis spectrophotometer (Thermofisher Scientific, Waltham, MA, USA) in a 96-well quartz glass plate (Hellma). For all spectra, the background was subtracted.

DC magnetic properties were investigated by means of a DC magnetometer (Lake Shore 7225, Lake Shore Cryotronics, Inc., Westerville, OH, USA) equipped with a cryogen-free magnet system at room temperature in quasistatic condition.

## 3. Results and Discussion

We performed two different synthesis methods—one was the conventional sol–gel synthesis and the other one was the coprecipitation method exploiting oleic acid as the stabilizing agent. The reaction rate of the sol–gel synthesis was very slow compared to that of the coprecipitation method. The sol–gel method took 6 h to synthesize Gd-doped ZnO-NCs and 18 h to synthesize Mn-doped ZnO-NCs. On the other hand, the coprecipitation method lasted only 10 min, irrespective of the dopant (Gd and Mn). In both the synthetic approaches, the color of Gd-doped ZnO-NC colloidal solutions was white, whereas the solutions of Mn-doped ZnO-NCs were light saffron in color.

### 3.1. Morphological and Structural Characterization

**Figure 1:**
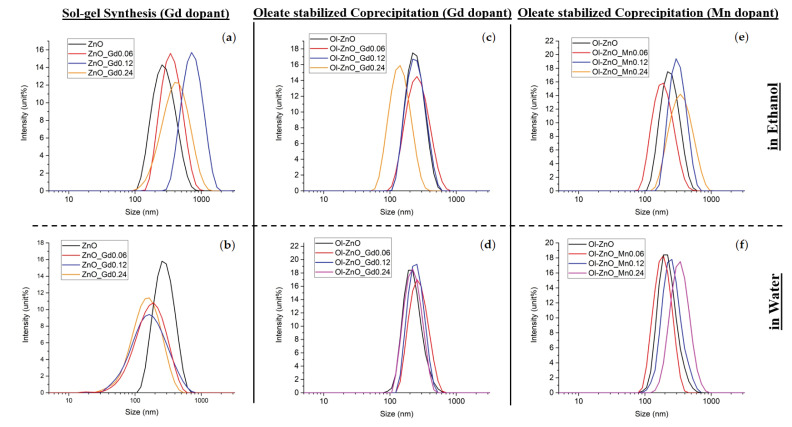
Dynamic light scattering (DLS) measurements in intensity % of Gd/Mn-doped ZnO-NCs synthesized by the traditional sol–gel method (**a**,**b**) and by oleic-acid-mediated coprecipitation method (**c**–**f**) for the (**c**,**d**) Gd dopant and the (**e**,**f**) Mn dopant. (**a**,**c**,**e**) Samples were analyzed in ethanol media; (**b**,**d**,**f**) samples were analyzed in water media.

The colloidal stability of the undoped and doped ZnO-NCs synthesized by both methods was evaluated by DLS and zeta potential measurements. All of the oleate-stabilized nanocrystals (undoped or Gd/Mn-doped) synthesized by coprecipitation method showed narrow size distributions and good dispersion in both ethanol and water media, having polydispersity indexes (PDI) of less than 0.36 (characteristic of monodisperse samples mostly, see Figure 1 and Table 1). On the other hand, Gd-doped ZnO-NCs synthesized by the traditional sol–gel method showed broad size distributions, especially in water media, all having comparable hydrodynamic diameters and polydispersity indexes (PDI) (Figure 1 and Table 1). Despite the undoped and Gd-doped nanocrystals, Mn-doped nanocrystals obtained via sol–gel synthesis showed very poor colloidal stability. The PDI values and hydrodynamic diameters were considerably higher in both ethanol and water media, indicating aggregation of the samples (Table 1). This can be explained by the longer reaction time (18 h) used for the synthesis of Mn-doped ZnO-NCs by the sol–gel method. As reported previously, increasing the reaction time may affect size or shape of NCs and change their surface structure, resulting in self-aggregation by oriented attachment of the nanoparticles [34,35]. We can assume that the surface properties of the Mn-doped samples synthesized by the sol–gel technique are not optimal in the used reaction condition, inducing heavy aggregation of the nanoparticles. Herein, we exploited oleic acid in chemical synthesis to stabilize the NCs against aggregation.

Additionally, along with the mean diameters of intensity-weighted distributions and PDI indexes, the zeta potential values for all the synthesized NCs are reported in Table 1.

**Figure 2:**
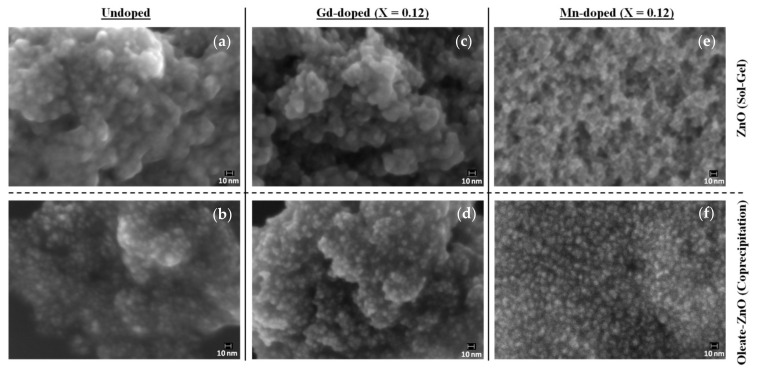
Field emission scanning electron microscopy (FESEM) images of undoped (**a**,**b**), Gd-doped (**c**,**d**), and Mn-doped (**e**,**f**) ZnO-NCs synthesized by either sol–gel method (**a**,**c**,**e**) or by oleic-acid-mediated coprecipitation method (**b**,**d**,**f**). The mole ratio of zinc acetate dihydrate with gadolinium acetate hydrate/manganese acetate tetrahydrate used for the synthesis was 1:0.12 (X).

With the aim of characterizing the shape and size of the NCs, we analyzed the samples using field emission scanning electron microscopy (FESEM). Except for Mn-doped nanocrystals synthesized by sol–gel synthesis, we obtained spherical-shaped, well-dispersed nanocrystals measuring approximately 5–8 nm in diameter (Figure 2 and Figure S1). Additionally, through the FESEM energy dispersive spectroscopic (EDS) analysis, we confirmed the presence of a doping element (Gd or Mn) in the NCs and evaluated its amount (Figures S2–S13). The atomic % of dopant concentration varied from 0.21 to 0.71 for Gd-doped ZnO-NCs synthesized by sol–gel method, from 0.17 to 0.53 for oleic-stabilized Gd-doped ZnO, and from 0.50 up to 1.51 for the Ol-ZnO–MnX samples (Figures S3–S8 and Figures S10–S12).

Since the aggregation of the ZnO–Mn0.12 sample was also confirmed by the FESEM analysis (Figure 2e and Figure S13), we excluded Mn-doped samples obtained by sol–gel synthesis for further analysis.

**Figure 3:**
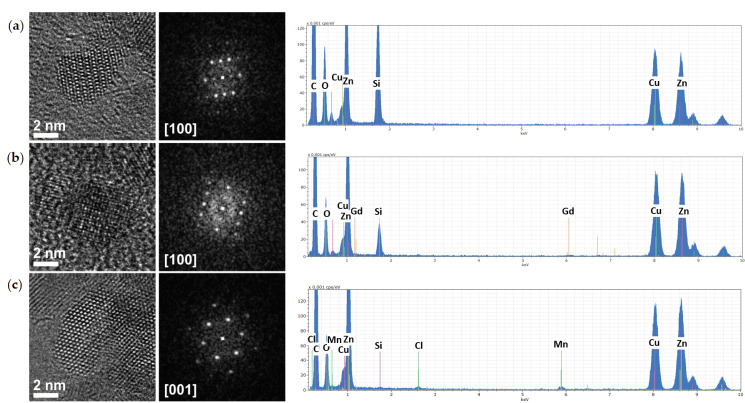
High-angle annular dark field-scanning transmission electron microscopy (HAADF-STEM, first panels from left), high-resolution transmission electron microscopy (HRTEM, second panels from left) images with respective Fast-Fourier Transform (FFTs, central panels) and scanning TEM coupled to energy dispersive spectra (STEM-EDS, first panels from right) analysis of Ol-ZnO (**a**), Ol-ZnO–Gd0.12 (**b**), and Ol-ZnO–Mn0.12 (**c**).

To confirm the shapes and sizes of the undoped and doped ZnO-NCs, as well as to study the crystallinity, HAADF-STEM and HRTEM imaging were further adopted. Figure 3 (left panels) shows the images of oleate-stabilized undoped and Gd/Mn-doped ZnO-NCs synthesized by the coprecipitation method, while Figure S14 shows the images for undoped and Gd-doped ZnO-NCs synthesized by sol–gel method. A rather isotropical shape was determined for the oleate-stabilized ZnO-NCs, with a diameter of about 6 nm (Figure 3). An isotropic shape and a diameter in the same range were observed for NCs synthesized by sol–gel method (Figure S14). Importantly, the isotropic shape is generally considered as the proper, biocompatible geometry for bio-applications. The presence of dopants (Gd or Mn) in the prepared samples was also confirmed by STEM-EDS mode (Figure 3 and Figure S14). Furthermore, a single-crystalline structure was observed for the NCs, and fast Fourier transforms (FFTs) of HRTEM images can be attributed to the wurtzite structure, which is the common crystalline structure of ZnO. This analysis does not show any indication of lattice distortion or expansion due to the doping, which may be due to the relatively low concentration of dopants used for these syntheses. We need also to consider the relatively low inherent accuracy of magnification calibration in HRTEM, which is generally not better than 5% [36], and the few degrees of uncertainty in the angles.

**Figure 4:**
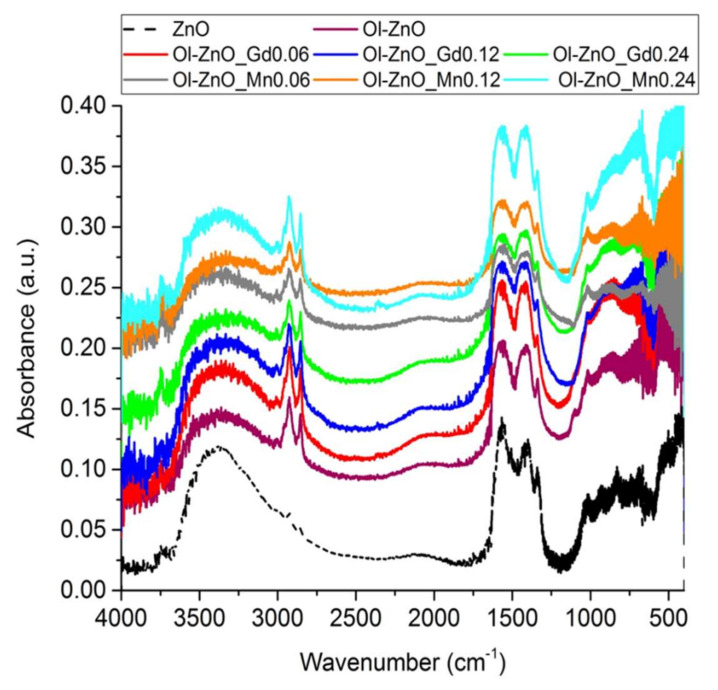
Fourier transform-infrared (FT-IR) spectra of ZnO-NCs synthesized by sol–gel method (dash line plot), and undoped and doped oleate-stabilized ZnO-NCs synthesized by coprecipitation method (solid line plots).

The presence of the oleate motif in undoped and Gd/Mn-doped ZnO-NCs synthesized by coprecipitation method was supported by the Fourier transform infrared (FT-IR) results. The FT-IR spectrum of ZnO-NCs synthesized by sol–gel method was also included as a reference. In general, all the FT-IR spectra (Figure 4) showed some common features, such as a broad band from 3600 to 3200 cm^−1^ corresponding to the stretching vibration of hydroxyl groups on the ZnO surface and an intense mode at ~440 cm^−1^ related to Zn–O vibration. Furthermore, the peaks at ~1570 and ~1420 cm^−1^ were attributed to the C=O and C–O vibrations, respectively, along with the peaks at ~2925 and ~2860 cm^−1^ concerning asymmetric and symmetric stretching vibrations of –CH_2_ and –CH_3_ groups, which corresponded to the residual acetate groups on the ZnO surface due to the precursors used or to the methoxy or ethoxy groups derived from the reflux conditions of the solvent used in the synthetic procedure [32]. Nevertheless, compared to the conventional ZnO-NCs, the stretching modes at ~2925 and ~2860 cm^−1^ are very intense for all the oleate-stabilized nanocrystals (undoped or Gd/Mn-doped) (Figure 4) due to the presence of –CH_2_ groups in the oleate motif [28], thus confirming the success of the coprecipitation method in synthesizing oleate-stabilized ZnO-NCs. Importantly, the peaks at 1725–1700 cm^−1^, related to the stretching vibrations of the C=O group of the free oleic acid, do not appear in the FT-IR spectra (Figure 4), suggesting formation of a monomolecular layer on the nanosized ZnO surface [28].

**Figure 5:**
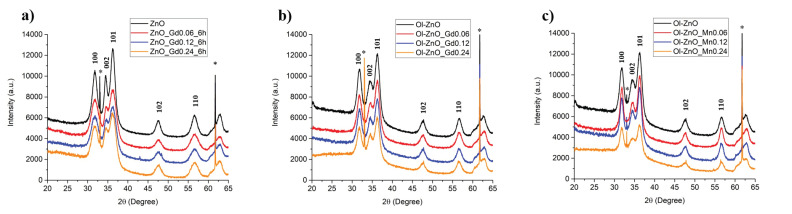
X-ray diffractograms of undoped and Gd-doped ZnO-NCs synthesized by traditional sol–gel method (ZnO–GdX–6h) (**a**), along with oleate-stabilized undoped and Gd-doped ZnO-NCs synthesized by coprecipitation method (Ol-ZnO–GdX) (**b**) and oleate-stabilized undoped and Mn-doped ZnO-NCs synthesized by coprecipitation method (Ol-ZnO–MnX) (**c**); X = 0, 0.06, 0.12, 0.24 (*silicon wafer peaks).

X-ray diffraction (XRD) measurements were carried out in order to determine the crystallinity of the NCs (Figure 5). The diffraction peaks of all the samples in XRD patterns can be explicitly indexed and allocated to the typical crystalline properties (wurtzitic structure) of ZnO, which has a hexagonal crystal system, in agreement with HRTEM. As there were no additional peaks appearing in the XRD patterns of the doped ZnO-NCs, we can exclude the formation of undesired oxides made by doping elements (Figure 5). In addition, the position shifts towards the lower 2*θ* angles of peaks (100), (002), and (101) for both the Gd-doped and Mn-doped samples with respect to undoped samples were analyzed. The variations of the 2theta positions (Δ2*θ* values) are reported in Table 2, suggesting increased lattice parameters upon doping.

Furthermore, the average crystallite size of each sample was calculated from the full width at half maximum (FWHM) of the strongest reflection (101) peak by using the Debye–Scherrer equation, *D* = kλ/*β*cos*θ*, as reported in Section 2.4, the values for which are shown in Table 3. The calculated mean crystallite diameters range from 3.9 nm to 7.5 nm, comparable to the sizes obtained by FESEM and HRTEM characterization, thus further confirming that the nanoparticles are single-crystalline in structure. Herein, all the Gd-doped samples have lower Debye–Scherrer diameters with respect to the corresponding undoped ZnO-NCs. This can plausibly be explained by the lattice contraction due to the hydrostatic pressure usually generated by the rare earth dopants situated on the surface of the ZnO-NCs [37]. On the other hand, the Debye–Scherrer diameters of the Mn-doped samples increase at low dopant concentrations, suggesting the incorporation of Mn ions (in the high-spin state) into the hexagonal wurtzite lattice of ZnO-NCs. At higher Mn doping concentrations (Ol-ZnO–Mn0.24), the decreased Debye–Scherrer diameter can be ascribed to the migration of Mn ions out of the wurtzite structure, accumulating on the ZnO-NC surface [38].

**Figure 6:**
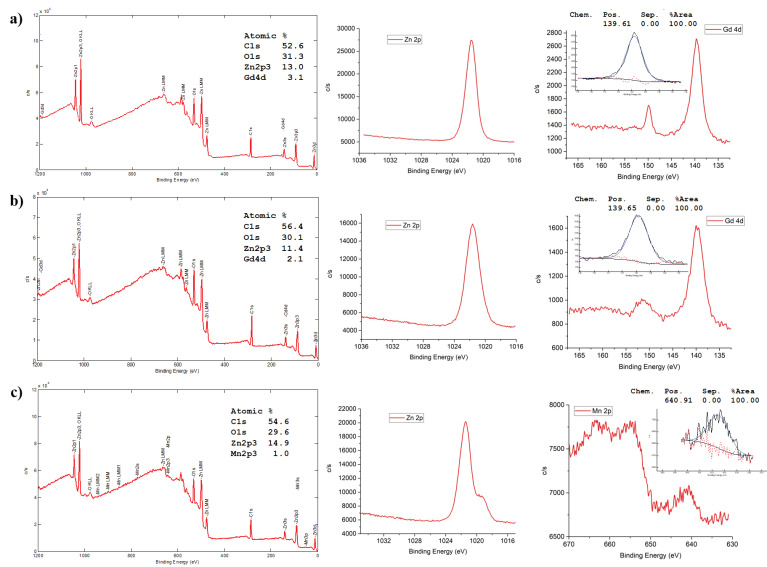
X-ray photoelectron spectroscopy (XPS) analysis of ZnO–Gd00.12 (**a**), Ol-ZnO–Gd0.12 (**b**), and Ol-ZnO–Mn0.12 (**c**). Survey spectra (left panels), Zn 2p high-resolution XPS spectra (central panels), dopant-specific high-resolution XPS spectra (right panels, Gd 4d are depicted in the first and second row, Mn 2p in the third row).

We also performed XPS analysis, a selective and sensitive technique for surface characterization, to determine the chemical compositions of the nanocrystals. Moreover, this technique is effective to monitor the characteristic binding energies (valence) to evaluate the constituent atoms (ions). The survey spectra of Gd/Mn-doped ZnO-NCs (in particular those having a 0.12 molar ratio of the dopant), mainly showing carbon, oxygen, zinc, and gadolinium/manganese (for doped NCs) species, were reported in Figure 6. Additionally, high-resolution spectra were recorded to determine the electronic states of the elements. In the Zn 2p high-resolution XPS spectrum, the peak at (1021.5 ± 0.1) eV refers to the binding energy of Zn 2p_3/2_. This is consistent with the emission of 2p photoelectrons from ZnO [33]. The shoulder of Zn 2p in Figure 5c is centered at 1019.27 eV and can be associated with the Zn broken bonds or O vacancies [39,40]. Additionally, the peak at 139.6 eV appearing in the Gd 4d spectra can be ascribed to the Gd4d_5/2_, showing that the oxidation state of Gd ions in the NPs is +3, which exist in the form of Gd_2_O_3_ [41]. The detectable peak of the Mn 2p spectrum centered at 640.9 eV can be attributed to the Mn 2p_3/2_, which is in good agreement with the binding energy of MnO [42]. Therefore, the above results further demonstrate the successful doping of Gd and Mn in ZnO-NCs.

To summarize, we can state that the nanocrystals synthesized via either conventional sol–gel synthesis or oleic-acid-mediated coprecipitation method have comparable structural and morphological characteristics when the dopant is Gd. On the other hand, although oleate-stabilized Mn-doped ZnO-NCs have comparable characteristics, the sol–gel method is not able to synthesize colloidally stable Mn-doped ZnO-NCs. Hence, we may conclude that the exploitation of oleic acid can facilitate the synthesis of high-quality doped ZnO-NCs, irrespective of the dopant used.

### 3.2. Optical and Magnetic Characterization

**Figure 7:**
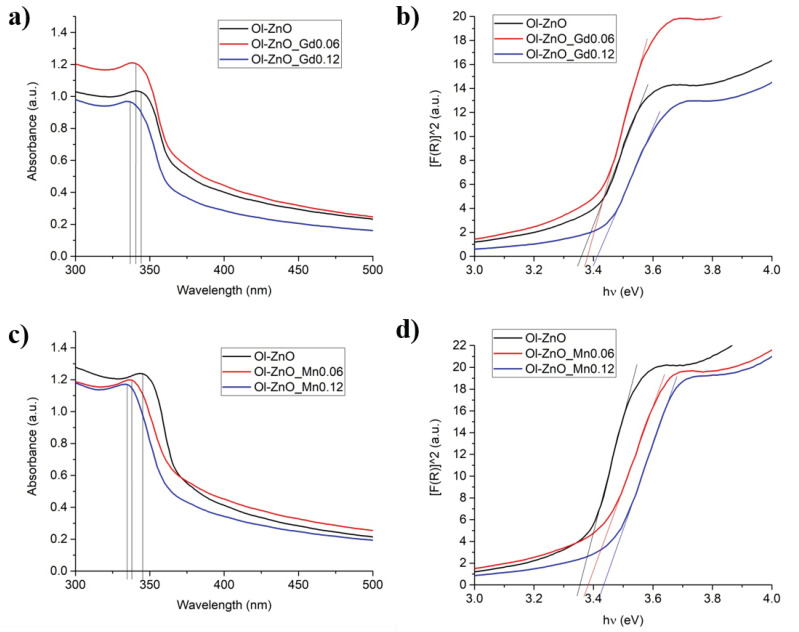
Characterization of the optical properties of Ol-ZnO–GdX (**a,b**) and Ol-ZnO–MnX (**c,d**) with different amounts of dopant (X = 0, 0.06 and 0.12): (**a,c**) ultraviolet–visible (UV−Vis) absorption spectra; (**b,d**) optical band gaps (*E*g).

We performed UV–Vis absorption spectroscopy in the region of 300–800 nm and determined the absorption wavelengths of the undoped and doped ZnO-NCs. For all samples, similar types of intense UV absorption were recorded only in the region of 300–350 nm, while there no bands were observed in the visible region (>450 nm) for the doped samples compared to that of undoped ZnO-NCs. This can be ascribed to the framework of the charge neutrality level and the concentration quenching process. Visible-range peaks are usually attributed to deep oxygen vacancy defects in ZnO-NCs. Introducing the dopant ions with higher positive charges (e.g., Gd^3+^, Mn^3+^, Mn^4+^) than the Zn^2+^ in ZnO-NCs increases the oxygen vacancy concentration, maintaining the charge neutrality in the crystal lattice. If the dopant concentration is low, the enhanced oxygen vacancies would not be adequate to expand the visible light absorption. Conversely, beyond the optimum concentrations of the dopants, the visible range peaks fade away, suggesting that the concentration quenching is related to aberrant defects and excess accumulation of dopants on the surface of ZnO-NCs [20,43,44]. Moreover, it was observed that the positions of the absorption spectra are shifted towards the lower wavelength at increasing dopant concentrations (Gd/Mn) (Figure 7 and Figure S15). This is normally known as blue-shift, correlating to the change in the optical band gap value. The optical band gaps (*E*g) of the samples were calculated using Tauc’s method from the absorption spectra, using the following formula: *E*g = hc/*λ*, where h is Planck’s constant (4.135667 × 10^−15^ eVs), c is the velocity of light (2.997924 × 108 m/s), and λ is the absorption wavelength (nm) [45], represented in Figure 7 and Figure S15. The values of the absorption wavelengths and band gaps obtained for undoped and doped ZnO-NCs are reported in Table 4 and Table S1. It can be assumed then that the blue shift in the bandgap is attributed to Gd or Mn doping in ZnO-NCs with the replacement of Zn in the ZnO lattice by either Gd or Mn. This reflects the increase in the band gap of ZnO nanocrystals with increasing doping ion concentration. This blue shift in the band gap can be explained by the phenomenon called the Burstein–Moss effect, where the change in bandgap is directly proportional to the carrier concentration. With the increase in doping concentration, the free charge carriers, which are donated by zinc interstitials and oxygen vacancies, increase. These free charge carriers eventually occupy the lowest state of the conduction band and cause the Fermi level to move to the conduction band. Consequently, the “apparent” band gap increases and is equivalent to the actual band gap + Moss–Burstein shift. As the optical transition of electrons can only take place from the valance band to the energy levels in the conduction band positioned above the Fermi level, the blue shift in the bandgap is obtained [18,25,46]. Additionally, from Figure 7 and Table 4, it is also clear that the higher increase of the band gap was observed for Ol-ZnO–Mn samples with respect to Ol-ZnO–Gd0 ones.

**Figure 8:**
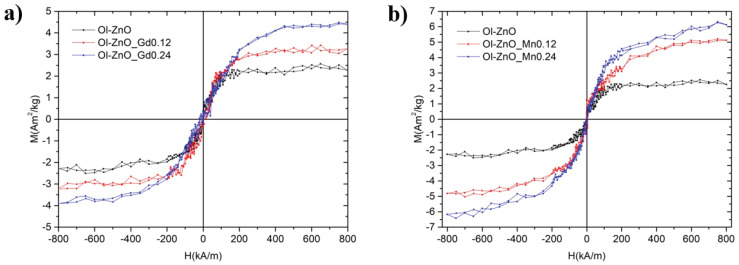
Magnetization-saturation (M–H) curves, showing the measured room Temperature (RT) magnetization curves for Ol-ZnO–GdX (**a**) and Ol-ZnO–MnX (**b**) with different amount of dopant (X = 0, 0.12, and 0.24).

DC magnetization measurements were performed on the undoped, Gd-doped, and Mn-doped oleate-stabilized ZnO-NCs, respectively, by cycling the applied magnetic field between ±800 kA/m at room temperature in quasistatic conditions (Figure 8). For comparison, the same magnetic measurements were performed on the Gd-doped ZnO synthesized by sol–gel method (Figure S16). The main effect on the magnetic behaviour from the introduction of Gd and Mn is the appearance of a paramagnetic signal, the magnitude of which is related to the amount of dopant. This result indicates the successful partial substitution of Gd and Mn atoms into the ZnO lattice. In particular, in the Gd-doped oleate-stabilized ZnO-NCs samples, the saturation magnetization varies from to 2.3 A·m^2^/kg of undoped sample up to 3.9 A·m^2^/kg (when X = 0.24). In the Mn-doped oleate-stabilized ZnO-NCs samples, the magnetization ranges from to 2.3 A·m^2^/kg (undoped sample) to 6.3 A·m^2^/kg (X = 0.24).

Recently, it has been reported that the observed weak ferromagnetic behaviour is switched into super paramagnetic behaviour by increasing the Gd concentration in the ZnO nanoparticles [47]. In any case, the magnetic behaviour in Gd-doped ZnO nanoparticles is caused by the exchange interactions among Gd and Zn ions [24]. Regarding the Gd-doped ZnO-NCs synthesized by sol–gel method, we detected a stronger magnetic effect (Figure S16) than with the oleate-stabilized doped ZnO-NCs. In particular, the highest saturation magnetization was 12.5 A·m^2^/kg (X = 0.12). This finding could be attributed to the overestimation of the Gd/Mn-doped NC mass in the oleate-stabilized sample due to the presence of oleic acid itself. Hence, it would not be justified to quantitatively compare the magnetic effects of the NCs synthesized by the two different methods. Regardless, we can confirm the presence of room-temperature ferromagnetism in all NCs synthesized both by sol–gel and oleate-based coprecipitation methods.

## 4. Conclusions

We report an easy and fast coprecipitation method exploiting oleic acid to synthesize nanosized and round-shaped Gd/Mn-doped ZnO-NCs and determine their room-temperature ferromagnetism. We also determine that the optical properties of ZnO nanocrystals are greatly improved using the optimum content of Mn/Gd doping. We show highly improved colloidal stability of oleate-stabilized Mn-doped ZnO-NCs compared to the Mn-doped ZnO-NCs synthesized by conventional sol–gel synthesis method, although both the synthesis methods work well for the Gd dopant. The oleate-stabilized coprecipitation method seems to be consistent, irrespective of the dopant, and can be expected to become the standard procedure used to synthesize either doped or codoped ZnO-NCs with any transition metal elements or rare earth elements in the future.

Taken together, the purpose of our work was to synthesize small-sized Gd/Mn-doped ZnO-NCs to improve the optical and magnetic properties of undoped ZnO-NCs by tuning the band gap using the optimum content of the dopant. Moreover, in the future, oleate-stabilized Gd/Mn-doped ZnO-NCs can be exploited as magnetic resonance imaging (MRI) contrast agents, as gadolinium and manganese elements can shorten the T1 or T2 relaxation time and eventually increase the signal intensity of T1-weighted images or reduce the signal intensity of T2-weighted images [48]. With these changes, we can expect a wide range of biomedical applications for the doped ZnO-NCs.

## Figures and Tables

**Table 1:** Hydrodynamic sizes (also called Z-Average, Z-Ave, size in both ethanol and water media), polydispersity indexes (PDI), and zeta potential values for the Gd/Mn-doped ZnO-NCs synthesized by traditional sol–gel method and by oleic-acid-mediated coprecipitation method.

Sample	Z-Ave Size (nm) (in Ethanol)	PDI	Z-Ave Size (nm) (in Water)	PDI	Zeta-Potential (mV) in Water
ZnO	256.7 ± 2.3	0.153 ± 0.03	268.9 ± 2.8	0.218 ± 0.01	22.0 ± 0.34
ZnO–Gd0.06	338.8 ± 4.3	0.143 ± 0.022	156.9 ± 6.4	0.344 ± 0.02	27.1 ± 0.82
ZnO–Gd0.12	381.7 ± 6.8	0.223 ± 0.01	196.7 ± 5.1	0.376 ± 0.01	26.8 ± 0.36
ZnO–Gd0.24	370.9 ± 5.8	0.169 ± 0.02	137.5 ± 2.9	0.333 ± 0.01	28.5 ± 0.69
ZnO–Mn0.06	3118 ± 557.0	0.837 ± 0.08	1962 ± 947.2	0.886 ± 0.19	28.2 ± 1.19
ZnO–Mn0.12	3629 ± 598.9	0.864 ± 0.12	1691 ± 829.5	0.835 ± 0.21	18.4 ± 1.34
ZnO–Mn0.24	3900 ± 844.0	0.835 ± 0.11	1727 ± 126.7	1.000 ± 0	22.2 ± 1.06
Ol-ZnO	227.4 ± 1.67	0.098 ± 0.02	232.9 ± 7.16	0.251 ± 0.04	18.9 ± 0.45
Ol-ZnO–Gd0.06	242.6 ± 6.06	0.138 ± 0.01	307.6 ± 3.98	0.318 ± 0.02	18.2 ± 0.35
Ol-ZnO–Gd0.12	236.0 ± 2.35	0.137 ± 0.01	286.5 ± 3.12	0.325 ± 0.01	17.7 ± 0.36
Ol-ZnO–Gd0.24	132.3 ± 0.55	0.102 ± 0.02	229.1 ± 3.25	0.357 ± 0.01	14.3 ± 0.10
Ol-ZnO–Mn0.06	179.0 ± 1.86	0.130 ± 0.01	219.5 ± 4.15	0.331 ± 0.07	17.4 ± 0.81
Ol-ZnO–Mn0.12	308.1 ± 4.31	0.120 ± 0.00	295.8 ± 4.33	0.314 ± 0.00	16.0 ± 0.11
Ol-ZnO–Mn0.24	351.0 ± 3.60	0.242 ± 0.00	359.4 ± 1.56	0.354 ± 0.04	15.7 ± 0.10

**Table 2:** Shift of X-ray reflection at Bragg angles (Δ2*θ*) for (100), (002), and (101). The shift calculations for the three diffraction maxima are based on each respective synthesis of ZnO without dopants.

	ZnO–Gd0.06	ZnO–Gd0.12	ZnO–Gd0.24	Ol-ZnO–Gd0.06	Ol-ZnO–Gd0.12	Ol-ZnO–Gd0.24	Ol-ZnO–Mn0.06	Ol-ZnO–Mn0.12	Ol-ZnO–Mn0.24
**Δ** **2*θ* (100)**	0.032	0.040	0.054	0.008	0.021	0.037	0.015	0.033	0.034
**Δ** **2*θ* (002)**	−0.020	0.009	0.051	0.016	0.021	0.036	0.009	0.028	0.055
**Δ** **2*θ* (101)**	0.029	0.035	0.046	0.013	0.032	0.049	0.002	0.007	0.035

**Table 3:** Debye–Scherrer diameters (nm) calculated from Full Width Half Maximum (FWHM) of the strongest reflection (101) peaks of undoped and Gd/Mn-doped ZnO-NCs.

	ZnO	ZnO–Gd0.06	ZnO–Gd0.12	ZnO–Gd0.24	Ol-ZnO	Ol-ZnO–Gd0.06	Ol-ZnO–Gd0.12	Ol-ZnO–Gd0.24	Ol-ZnO–Mn0.06	Ol-ZnO–Mn0.12	Ol-ZnO–Mn0.24
Debye–Scherrer diameters (nm)	5.70	3.92	3.98	4.10	5.98	5.63	5.70	5.62	7.41	6.65	5.83

**Table 4:** Analyzed absorption wavelength (*λ*) and band gap values from UV−Vis absorption spectra of oleate-stabilized undoped and Gd/Mn-doped ZnO-NCs.

	Ol-ZnO	Ol-ZnO–Gd0.06	Ol-ZnO–Gd0.12	Ol-ZnO	Ol-ZnO–Mn0.06	Ol-ZnO–Mn0.12
*λ* (nm)	344.09	340.63	336.75	345.28	337.89	334.93
Band gap (*E*g)	3.36	3.38	3.41	3.35	3.38	3.43

## Supplementary Materials

The following are available online at https://www.mdpi.com/2079-4991/10/6/1150/s1, Figure S1: Additional Field emission scanning electron microscopy (FESEM) images of Gd-doped and Mn-doped ZnO-NCs, Figures S2–S13: FESEM-Energy Dispersive Spectroscopic (EDS) analysis of Gd-doped and Mn-doped ZnO-NCs, Figure S14: HAADF-STEM, HRTEM images with respective FFTs and STEM-EDS analysis of ZnO and ZnO_Gd0.24. Figure S15: Characterization of the optical properties of Gd-doped ZnO-NCs, Figure S16. Magnetization curves for Gd-doped ZnO-NCs synthesized by Sol-gel method. Table S1: absorption wavelength and band gap values from UV−vis absorption spectra of Gd-doped ZnO-NCs.
