# Supplementary material for: Facile Chemical Synthesis of Doped ZnO Nanocrystals Exploiting Oleic Acid
Source: Nanomaterials (Basel). 2020 Jun 11;10(6):1150. doi: 10.3390/nano10061150 (PMC7353420; doi:10.3390/nano10061150)
Supplement: Supplementary file 1 [file nanomaterials-10-01150-s001.pdf]

Supporting Information

Facile chemical synthesis of doped ZnO nanocrystals exploiting oleic acid

Sugata Barui[1], Roberto Gerbaldo[1], Nadia Garino[1], Rosaria Brescia[2], Francesco Laviano[1], Valentina Cauda[1, *]

[1] Department of Applied Science and Technology, Politecnico di Torino, Corso Duca degli Abruzzi 24, 10129 Turin, Italy; sugata.barui@polito.it; roberto.gerbaldo@polito.it; nadia.garino@polito.it; francesco.laviano@polito.it

[2] Electron Microscopy Facility, Center for Convergent Technologies, Istituto Italiano di Tecnologia (IIT), via Morego 30, 16163 Genoa, Italy; rosaria.brescia@iit.it

* Correspondence: valentina.cauda@polito.it; Tel.: +39-011 090 7389

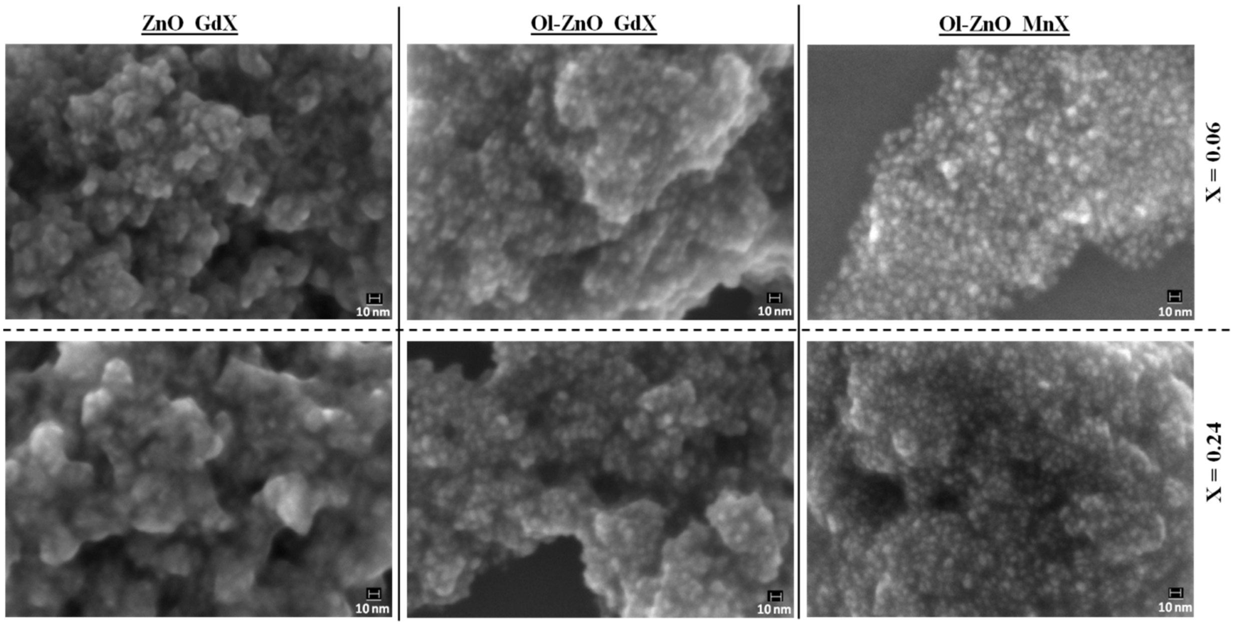

Figure S1. Field emission scanning electron microscopy (FESEM) images of Gd-doped ZnO-NCs synthesized either by Sol-gel method (ZnO_GdX; left panels) or by oleic acid mediated coprecipitation method (Ol-ZnO_GdX; middle panels) and Mn-doped ZnO-NCs synthesized by oleic acid mediated coprecipitation method (Ol-ZnO_MnX; right panels) where X = 0.06, 0.24.

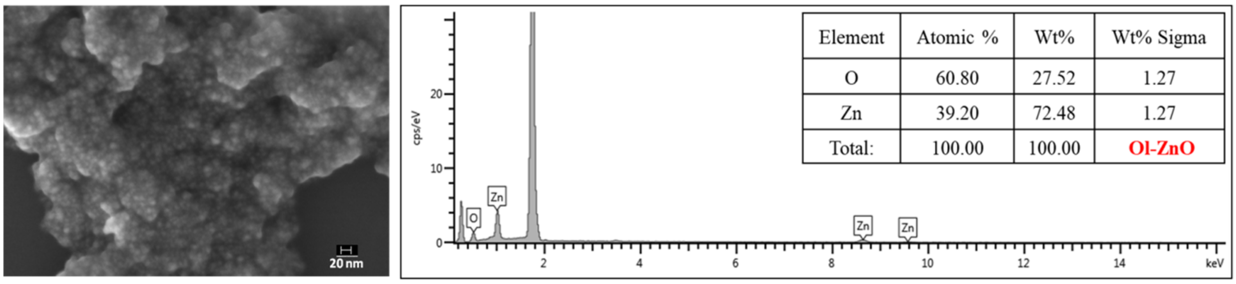

Element	Atomic %	Wt%	Wt% Sigma
O	60.80	27.52	1.27
Zn	39.20	72.48	1.27
Total:	100.00	100.00	Ol-ZnO

Figure S2. FESEM-Energy Dispersive Spectroscopic (EDS) analysis of Ol-ZnO NCs.

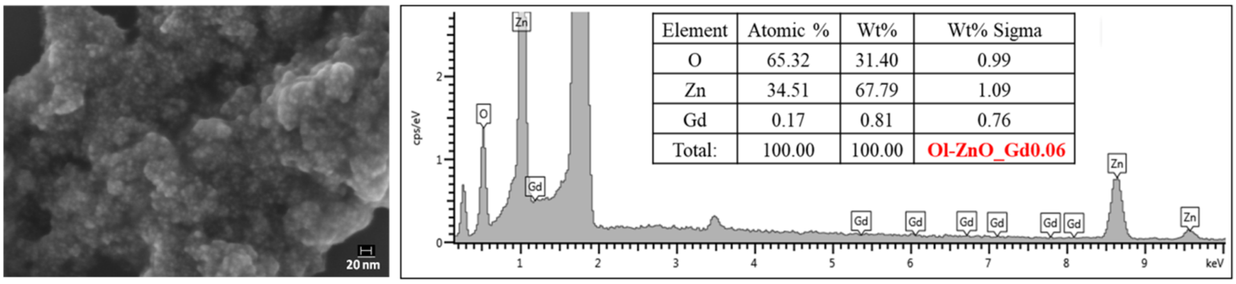

Element	Atomic %	Wt%	Wt% Sigma
O	65.32	31.40	0.99
Zn	34.51	67.79	1.09
Gd	0.17	0.81	0.76
Total:	100.00	100.00	Ol-ZnO_Gd0.06

Figure S3. FESEM-Energy Dispersive Spectroscopic (EDS) analysis of Ol-ZnO_Gd0.06.

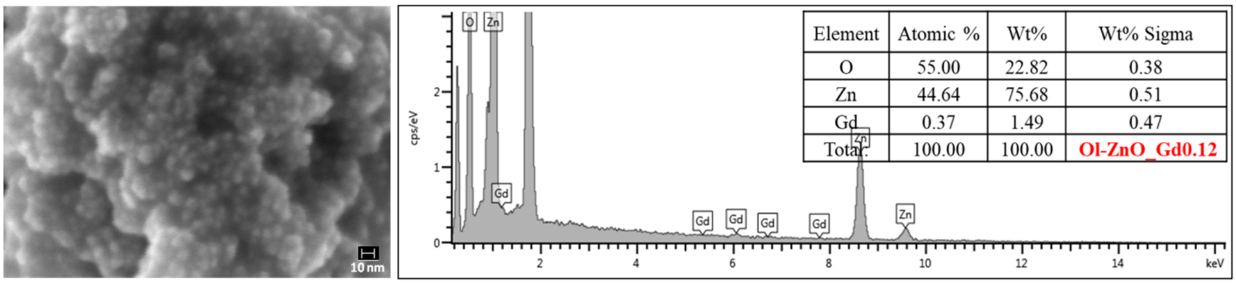

Element	Atomic %	Wt%	Wt% Sigma
O	55.00	22.82	0.38
Zn	44.64	75.68	0.51
Gd	0.37	1.49	0.47
Total:	100.00	100.00	Ol-ZnO_Gd0.12

Figure S4. FESEM-Energy Dispersive Spectroscopic (EDS) analysis of Ol-ZnO_Gd0.12.

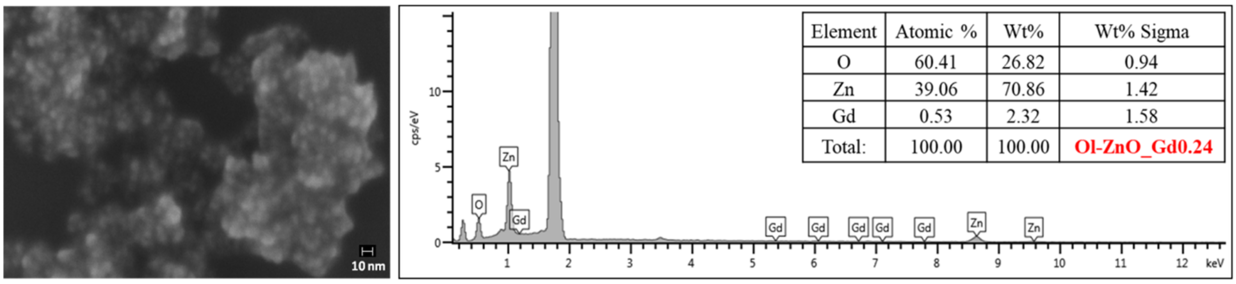

Element	Atomic %	Wt%	Wt% Sigma
O	60.41	26.82	0.94
Zn	39.06	70.86	1.42
Gd	0.53	2.32	1.58
Total:	100.00	100.00	Ol-ZnO_Gd0.24

Figure S5. FESEM-Energy Dispersive Spectroscopic (EDS) analysis of Ol-ZnO_Gd0.24.

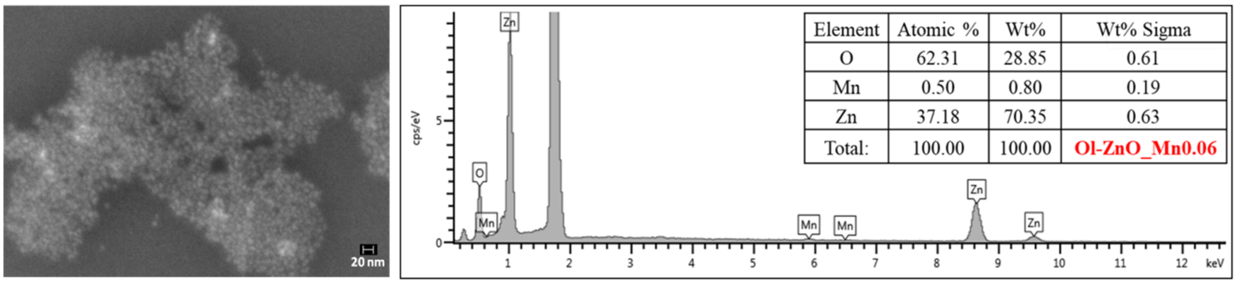

Element	Atomic %	Wt%	Wt% Sigma
O	62.31	28.85	0.61
Mn	0.50	0.80	0.19
Zn	37.18	70.35	0.63
Total:	100.00	100.00	Ol-ZnO_Mn0.06

Figure S6. FESEM-Energy Dispersive Spectroscopic (EDS) analysis of Ol-ZnO_Mn0.06.

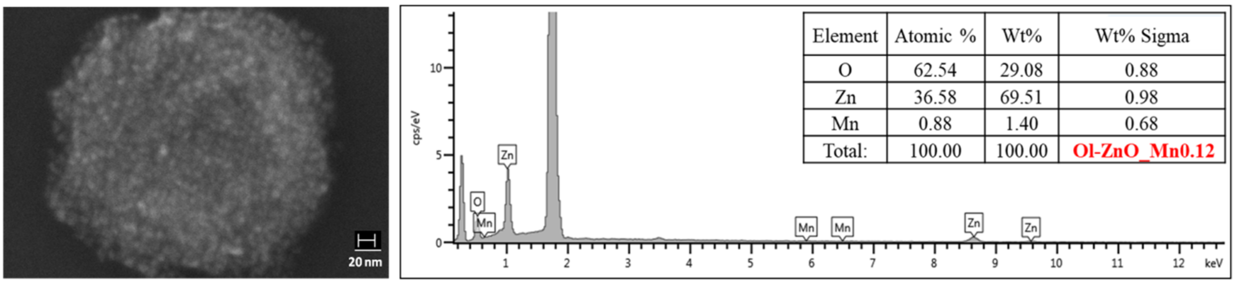

Element	Atomic %	Wt%	Wt% Sigma
O	62.54	29.08	0.88
Zn	36.58	69.51	0.98
Mn	0.88	1.40	0.68
Total:	100.00	100.00	Ol-ZnO_Mn0.12

Figure S7. FESEM-Energy Dispersive Spectroscopic (EDS) analysis of Ol-ZnO_Mn0.12.

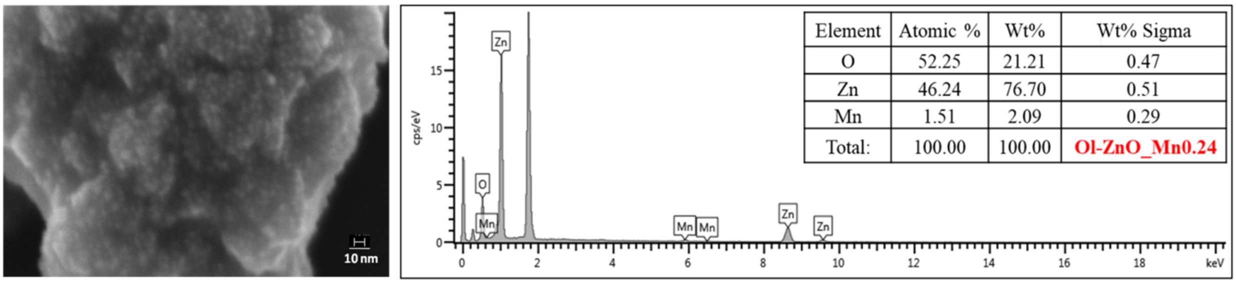

Element	Atomic %	Wt%	Wt% Sigma
O	52.25	21.21	0.47
Zn	46.24	76.70	0.51
Mn	1.51	2.09	0.29
Total:	100.00	100.00	Ol-ZnO_Mn0.24

Figure S8. FESEM-Energy Dispersive Spectroscopic (EDS) analysis of Ol-ZnO_Mn0.24.

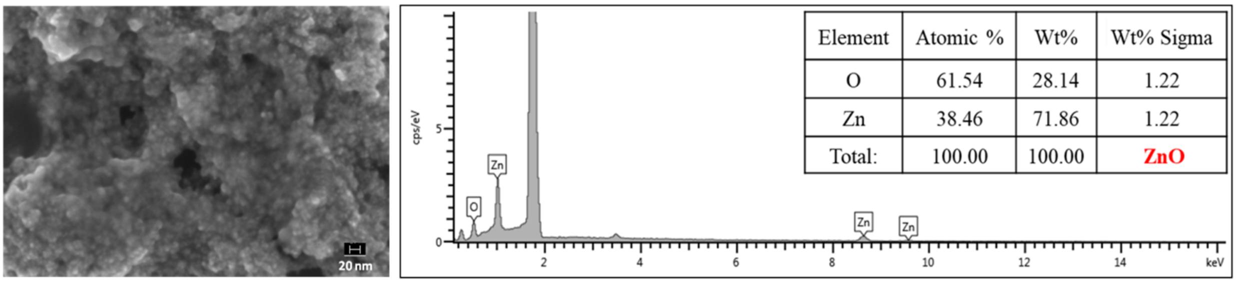

Element	Atomic %	Wt%	Wt% Sigma
O	61.54	28.14	1.22
Zn	38.46	71.86	1.22
Total:	100.00	100.00	ZnO

Figure S9. FESEM-Energy Dispersive Spectroscopic (EDS) analysis of ZnO-NCs.

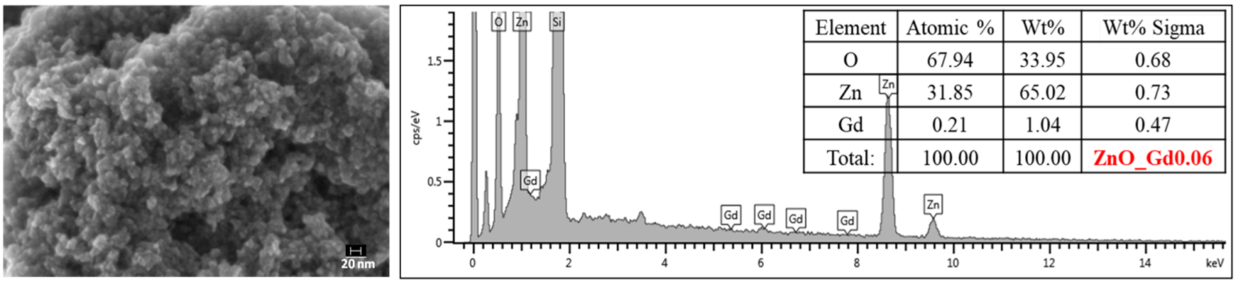

Element	Atomic %	Wt%	Wt% Sigma
O	67.94	33.95	0.68
Zn	31.85	65.02	0.73
Gd	0.21	1.04	0.47
Total:	100.00	100.00	ZnO_Gd0.06

Figure S10. FESEM-Energy Dispersive Spectroscopic (EDS) analysis of ZnO_Gd0.06.

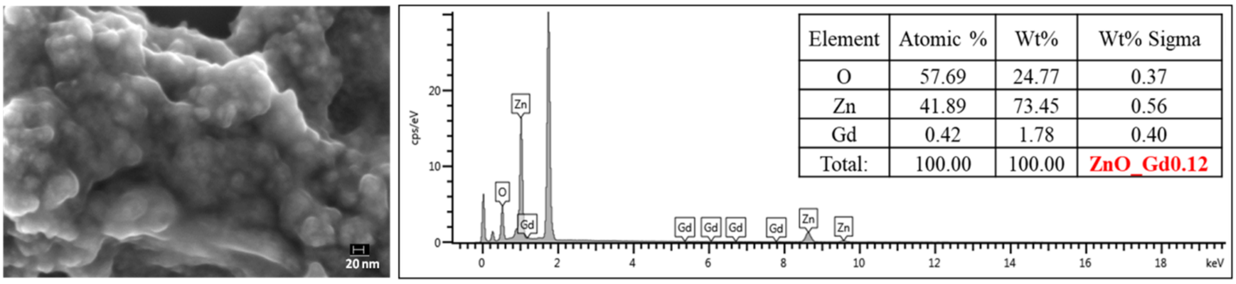

Element	Atomic %	Wt%	Wt% Sigma
O	57.69	24.77	0.37
Zn	41.89	73.45	0.56
Gd	0.42	1.78	0.40
Total:	100.00	100.00	ZnO_Gd0.12

Figure S11. FESEM-Energy Dispersive Spectroscopic (EDS) analysis of ZnO_Gd0.12.

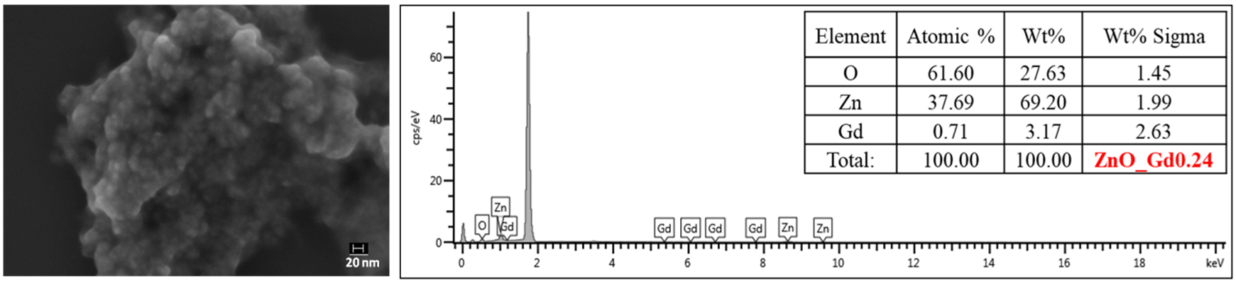

Element	Atomic %	Wt%	Wt% Sigma
O	61.60	27.63	1.45
Zn	37.69	69.20	1.99
Gd	0.71	3.17	2.63
Total:	100.00	100.00	ZnO_Gd0.24

Figure S12. FESEM-Energy Dispersive Spectroscopic (EDS) analysis of ZnO_Gd0.24.

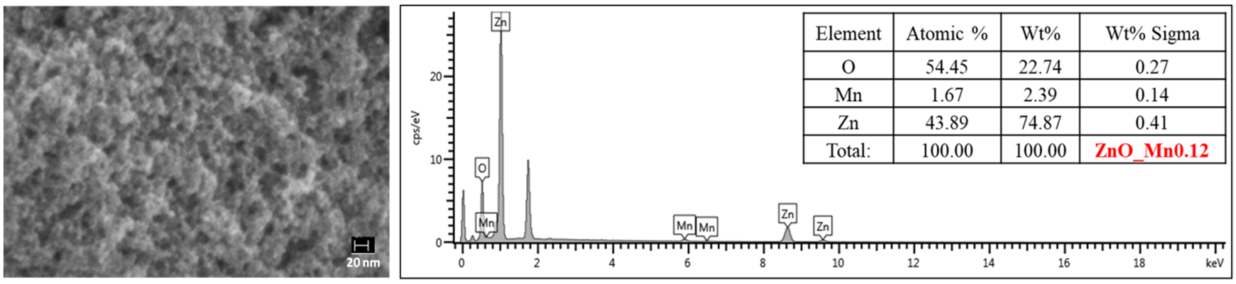

Element	Atomic %	Wt%	Wt% Sigma
O	54.45	22.74	0.27
Mn	1.67	2.39	0.14
Zn	43.89	74.87	0.41
Total:	100.00	100.00	ZnO_Mn0.12

Figure S13. FESEM-Energy Dispersive Spectroscopic (EDS) analysis of ZnO_Mn0.12.

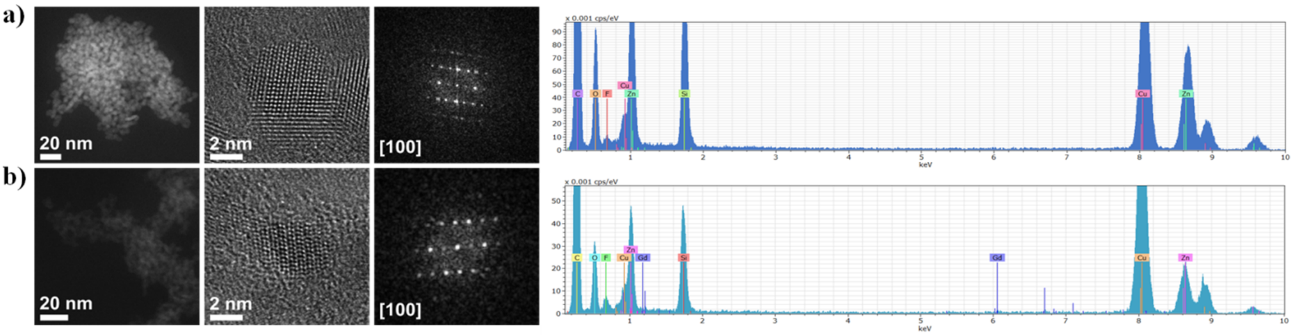

Figure S14. HAADF-STEM, HRTEM images with respective FFTs and STEM-EDS analysis of ZnO (**a**) and ZnO_Gd0.24 (**b**).

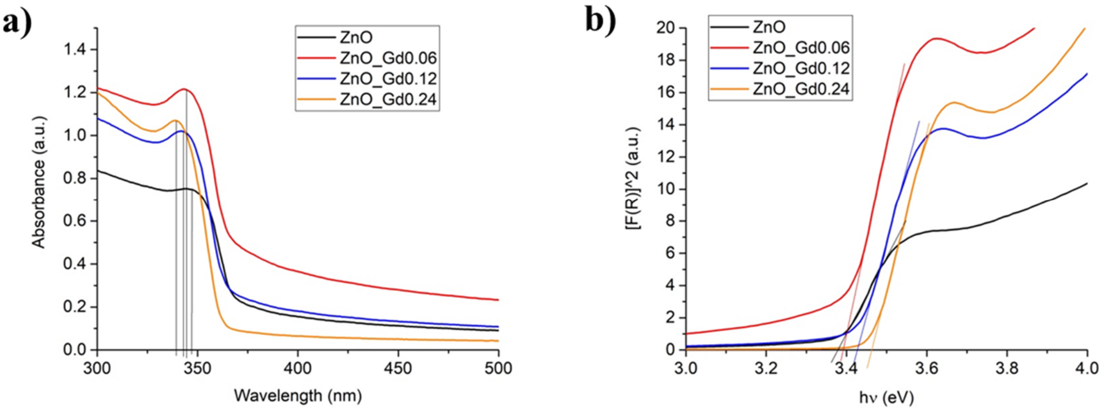

Figure S15. Characterization of the optical properties of Gd- doped ZnO-NCs synthesized by Sol-gel method (ZnO_GdX where X = 0, 0.06, 0.12 and 0.24). (**a**) represents Ultraviolet–visible (UV–vis) absorption spectra and (**b**) represents optical band gaps (Eg).

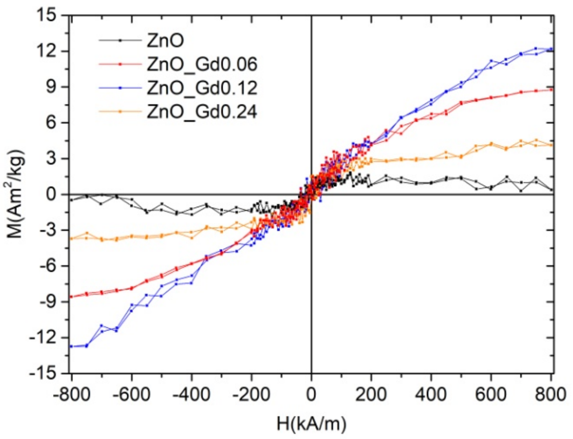

Figure S16. M-H curve, the measured RT magnetization curves for Gd- doped ZnO-NCs synthesized by Sol-gel method (ZnO_GdX where X = 0, 0.06, 0.12 and 0.24).

Table S1. Analyzed absorption wavelength (λ) and band gap values from UV−vis absorption spectra of Gd-doped ZnO-NCs synthesized by Sol-gel method.

	**ZnO**	**ZnO_Gd0.06**	**ZnO_Gd0.12**	**ZnO_Gd0.24**
**λ (nm)**	347.60	344.64	342.86	339.31
**Band gap (Eg)**	3.37	3.39	3.43	3.46
